# The evolution of fruit colour: phylogeny, abiotic factors and the role of mutualists

**DOI:** 10.1038/s41598-018-32604-x

**Published:** 2018-09-24

**Authors:** Kim Valenta, Urs Kalbitzer, Diary Razafimandimby, Patrick Omeja, Manfred Ayasse, Colin A. Chapman, Omer Nevo

**Affiliations:** 1 Duke University, Department of Evolutionary Anthropology, 130 Science Dr., Durham, NC 27708 USA; 2 McGill University, McGill School of the Environment and Department of Anthropology, 3534 University Ave., Montreal, Quebec H3A-2A7 Canada; 3 Faculty of Sciences, Zoology and Animal Biodiversity, University of Antananarivo, Antananarivo, Madagascar; 4 Makerere University Biological Field Station, P.O. Box 907, Fort Portal, Uganda; 5 University of Ulm, Institute of Evolutionary Ecology and Conservation Genomics, Albert-Einstein-Allee 11, Ulm, 89081 Germany

## Abstract

The adaptive significance of fruit colour has been investigated for over a century. While colour can fulfil various functions, the most commonly tested hypothesis is that it has evolved to increase fruit visual conspicuousness and thus promote detection and consumption by seed dispersing animals. However, fruit colour is a complex trait which is subjected to various constraints and selection pressures. As a result, the effect of animal selection on fruit colour are often difficult to identify, and several studies have failed to detect it. Here, we employ an integrative approach to examine what drives variation in fruit colour. We quantified the colour of ripe fruit and mature leaves of 97 tropical plant species from three study sites in Madagascar and Uganda. We used phylogenetically controlled models to estimate the roles of phylogeny, abiotic factors, and dispersal mode on fruit colour variation. Our results show that, independent of phylogeny and leaf coloration, mammal dispersed fruits are greener than bird dispersed fruits, while the latter are redder than the former. In addition, fruit colour does not correlate with leaf colour in the visible spectrum, but fruit reflection in the ultraviolet area of the spectrum is strongly correlated with leaf reflectance, emphasizing the role of abiotic factors in determining fruit colour. These results demonstrate that fruit colour is affected by both animal sensory ecology and abiotic factors and highlight the importance of an integrative approach which controls for the relevant confounding factors.

## Introduction

Understanding the origin and adaptive significance of fruit colour has been a lively source of debate for over a century^1–3^. While less varied than flower colour globally, fruit colour diversity is nonetheless extensive, spanning and surpassing the human capacity to detect it^4^. Fruit colour diversity has been attributed to phylogenetic constraints, environmental constraints, and protection from antagonists^1,4–6^. Yet the oldest, best documented, and most contentious hypothesis for why fruit colour is so diverse centres on its role in attracting seed dispersing mutualists^7^. The disperser hypothesis posits that the colour of fleshy fruits evolved to maximise visual detection by specific animal mutualists to facilitate seed dispersal^8,9^.

Dispersers differ markedly in their visual capacities: birds possess tetrachromatic colour vision^10^. Most mammals are dichromatic, and primates - a major seed disperser in tropical systems – are either dichromats, trichromats or polymorphic (i.e. individuals are either di- or trichromats)^11^. Moreover, frugivores differ in their activity patterns^12^ and tendency to rely on non-visual fruit signals and cues^13–16^. Thus, the disperser hypothesis also predicts that fruits of plant species that rely on dispersal by different frugivores are subject to selective pressures that differ in both their magnitude and direction^17,18^, and would result in different fruit colour.

Despite the breadth of research regarding fruit colour as an adaptation to attracting mutualists, the theory remains highly contentious, and evidence for it is mixed^17,19–21^. The ongoing disagreement regarding the adaptive significance of fruit colour diversity may partly stem from the fact that many studies of fruit colour have relied on subjective, human assessments of fruit colour, which means that species are assigned to categories like red, or yellow^3^. Efforts to assess forces and constraints shaping fruit colour variation that rely on subjective human categories thus *de facto* underestimate the diversity of fruit colour, and further, are likely to miscategorise fruit colours. For example, a fruit categorised as “black” may in fact be reflecting strongly in the ultraviolet (UV) – a range of reflectance that is visually salient to many birds, but invisible to humans^22^.

In addition to the methodological limitations of many studies that aim to understand fruit colour diversity, the number of potential variables affecting fruit colour further impedes efforts to understand its origin and significance. Fruit colour is likely driven by multiple variables including environmental, physiological, and phylogenetic constraints, in addition to the potential selection for maximizing detectability to dispersers^23^. More specifically, it has been proposed that various factors that affect leaf colour such as latitude, temperature, and soil properties may also affect fruit colour^6^. Thus, even when fruit colour is quantified, the potential importance of multiple predictive variables requires an approach that includes the effects of each variable in light of the effects of all relevant variables, including phylogeny, and the potential role of abiotic factors.

**Figure 1:**
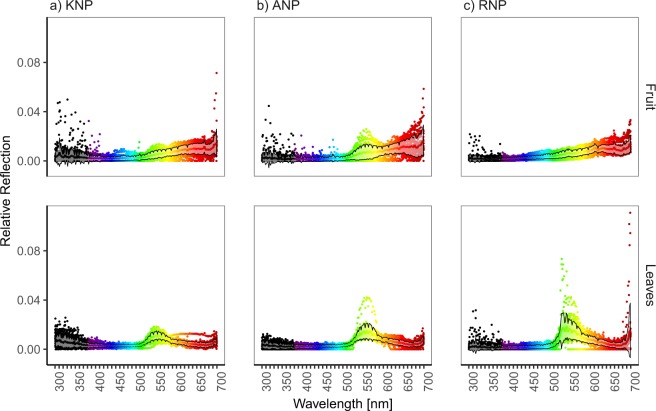
Mean fruit and leaf colour reflectance between 300–700 nm for (**a**) Kibale, (**b**) Ankarafantsika, and (**c**) Ranomafana National Parks. Reflectance values were summarised into 2 nm bins and the sum of all values per species is 1.

Here, we quantify fruit and leaf colour using spectrometric measurements, and apply a comparative approach to examine which factors drive fruit colour variation. We test three hypotheses regarding the source of fruit colour variation, using fruit colour spectra from three tropical systems: (1) Fruit colour is driven by phylogeny. (2) Fruit colour is in fact “plant colour” and is driven by constraints or adaptive response to abiotic factors. If fruit colour is primarily a response to such factors, fruit reflectance should resemble leaf reflectance (3) Fruit colour differs between dispersal syndromes. If fruit colour is under selection to maximise detectability to seed dispersers, plants that rely on frugivores with different visual phenotypes (mammals, birds) or tendency to rely on visual cues will produce fruits which are, on average, differently coloured. Using reflectance samples from ripe fruits and leaves of 97 plant species (Fig. 1), we calculate Phylogenetic Generalised Least Squares models (PGLS) models to test the effects of phylogeny, dispersal mode (mammal, bird, and mixed), and leaf colour on fruit colour, summarised in four variables corresponding to relative reflectance in four colour bands: UV (300–400 nm), blue (400–500 nm), green (500–600 nm), and red (600–700 nm). Crucially, since these three hypotheses are not mutually exclusive, our models include all three to control for their effects and thus identify the independent effects of each factor alone.

## Results

### The effect of phylogeny

We found no phylogenetic signal in fruit colour in any of the four reflectance bands (UV 300–400 nm; blue 400–500 nm; green 500–600 nm; red 600–700 nm. In all cases lambda <0.001; p = 1).

### Leaf colour

Of the four potential pairings of fruit and leaf colour, reflectance in the UV part of the spectrum (300–400 nm) in fruits was strongly positively related with UV reflectance in leaves, independent of phylogeny, dispersal syndrome, and site (Table 1). There was no such an effect for blue (Table 2), green (Table 3), or red (Table 4) reflectance.

**Table 1:** UV reflectance in fruits.

Term	Estimate	SE	df	*x* ^2^	P
(Intercept)	0.065	0.103			
UV in leaves	0.560	0.103	1	27.142	<**0**.**001**
Dispersal – Bird	0.027	0.042	2	2.136	0.344
Dispersal – Mixed	−0.028	0.032			
Site – KNP	0.017	0.038	2	3.826	0.148
Site – RNP	0.061	0.036			

Results of a PGLS model with square-root transformed reflectance values as response variable. The full model was significantly better than the null model (X^2^ = 32.856, df = 9, p < 0.001). The interaction between syndrome and site was not significant (Χ^2^ = 2.491, df = 4, P = 0.646) and was thus removed from the model in order to establish P-values for the main effects.

**Table 2:** Blue reflectance in fruits.

Term	Estimate	SE	df	*x* ^2^	p
(Intercept)	0.229	0.097			
Blue in leaves	0.079	0.113	1	0.524	0.469
Dispersal – Bird	−0.048	0.040	2	3.625	0.163
Dispersal – Mixed	−0.052	0.029			
KNP	0.057	0.034	2	9.550	<**0**.**01**
RNP	0.104	0.034			

Results of a PGLS model with square-root transformed reflectance values as response variable. The full model was significantly better than the null model (X^2^ = 21.614, df = 9, p < 0.05). The interaction between syndrome and site was not significant (Χ^2^ = 4.741, df = 4, P = 0.315) and was thus removed from the model in order to establish P-values for the main effects.

**Table 3:** Green reflectance in fruits.

Term	Estimate	SE	df	*x* ^2^	p
(Intercept)	0.436	0.125			
Green in leaves	0.170	0.124	1	1.965	0.161
Dispersal – Bird	−0.130	0.038	2	20.678	<**0**.**001**
Dispersal – Mixed	−0.125	0.029			
KNP	0.028	0.033	2	0.861	0.650
RNP	0.011	0.033			

Results of a PGLS model with square-root transformed reflectance values as response variable. The full model was significantly better than the null model (X^2^ = 30.618, df = 9, p < 0.001). The interaction between syndrome and site was not significant (Χ^2^ = 2.149, df = 4, P = 0.708) and was thus removed from the model in order to establish P-values for the main effects.

**Table 4:** Red reflectance in fruits.

Term	Estimate	SE	df	*x* ^2^	p
(Intercept)	0.774	0.117			
Red in leaves	0.169	0.156	1	1.239	0.266
Dispersal – Bird	0.103	0.039	2	12.330	<**0**.**01**
Dispersal – Mixed	0.095	0.030			
KNP	−0.059	0.035	2	2.971	0.226
RNP	−0.046	0.034			

Results of a PGLS model with square-root transformed reflectance values as response variable. The full model was significantly better than the null model (X^2^ = 21.190, df = 9, p < 0.05). The interaction between syndrome and site was not significant (Χ^2^ = 3.686, df = 4, P = 0.450) and was thus removed from the model in order to establish P-values for the main effects.

### The effect of dispersers

**Figure 2:**
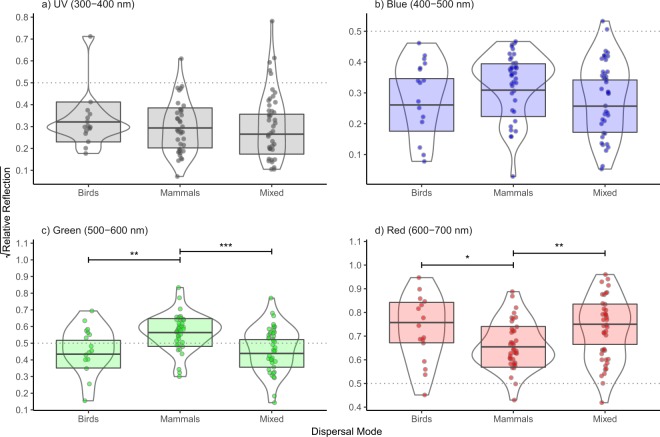
Relative reflectance of fruit by dispersal mode. Horizontal bars indicate significant differences in reflectance between different dispersal categories. *<0.05, **<0.01, ***<0.001 (P values were adjusted using the Tukey method). The shaded boxes with the horizontal bars show the predicted means and standard errors of the PGLS models, which were estimated using the emmeans package in R^63^, and which were averaged over the levels of the categorical predictor variable site and assuming an average value for the numerical predictor variable leave reflectance in the respective colour band. The dashed grey lines at 0.5 root-squared relative reflectance were added to facilitate the comparison of the four plots.

Because the visual phenotypes of mammals and birds can differ between different sites, we first examined whether the relationships between dispersal mode and fruit colour varied by site by including the interaction between dispersal mode and site. This interaction was not significant in any of the colour bands (Tables 1–4), implying that the effect of dispersal syndrome is consistent across sites. After removing the interaction terms from the models, we also did not detect a significant main effect of dispersal mode on reflectance in the UV (Table 1) or blue (Table 2) parts of the spectrum. However, in the green and red colour bands, dispersal mode showed significant relationships with reflectance (Tables 3 and 4). Post-hoc tests revealed that mammal-dispersed fruits reflected significantly more than either bird-dispersed or mixed-dispersed fruits in the green part of the spectrum, and significantly less in the red parts of the spectrum (Fig. 2).

## Discussion

We tested three non-mutually-exclusive hypotheses for fruit colour variation; that fruit colour is (1) phylogenetically constrained, (2) a response to abiotic factors, and (3) adapted to seed dispersing mutualists. We found no evidence to support the hypothesis that fruit colour is the result of phylogenetic constraints, but we found evidence for the importance of environmental factors, and dispersers in fruit colour variation.

Our finding that fruit colour is not driven by phylogenetic constraints corroborates several recent studies showing that fruit traits, such as scent and size, can be malleable to selection pressures exerted by animal mutualists^4,8,16,20,24,25^, and contradicts the longstanding hypothesis that fruit traits are strongly conserved^26,27^. The finding that fruit and leaf reflectance in only the UV part of the spectrum are correlated may indicate the importance of solar radiation in fruit colour, at least across certain parts of the spectrum. While solar radiation is required to maintain plant function, excess light absorption can be damaging, and even fatal, to plant tissues^28^, which may result in the use of plant pigments as photoprotective screens^29^. Absorption at certain spectra, particularly in the ultraviolet, can result in photoinhibition and photodamage – reduced photosynthetic efficiency and cell damage^30^. Plants have mechanisms to detect and respond to variation in ambient light, and to attenuate harmful solar radiation via investment in compounds, like red-reflecting anthocyanins^31^. While understudied in fruits relative to leaves, studies have documented increased fruit investment in anthocyanins in *Vaccinium* species along altitudinal and latitudinal gradients^32,33^, and that UV reflection in leaves and fruits of this genus are correlated^34^ indicating that photoprotection may also be important in fleshy fruits. Plant UV reflectance may also function to reduce water loss in plant parts^35^. Experimentally, UV reflectance has been found to increase likelihood of ripening, and responsive to both solar radiation and edaphic factors in temperate, agricultural systems^36^. Increased solar radiation may result from latitudinal and altitudinal differences in solar radiation^33^, a plant’s position in the canopy relative to conspecifics^37^, and seasonality^38^. If plant mechanisms for UV reflectance are labile, this has potential implications for fruit colour and plant-animal mutualisms as spatio-temporal patterns in solar radiation are altered by climate change^39^. While beyond the scope of this study, future studies can test whether UV reflectance in fruits or leaves is associated with abiotic conditions such as growth form, elevation, and latitude.

Our results demonstrate that when colour is quantitatively measured rather than qualitatively described, and other potential factors are controlled for, the hypothesis that fruit colour is associated with seed dispersing mutualists is supported in these systems. We found clear differences in fruit coloration between species that rely solely on mammals for seed dispersal and those that rely, at least partially, on birds. Bird- and mixed-dispersed fruits reflect more in the red part of the spectrum, while mammal-dispersed fruits reflect more in the green part of the spectrum. Fruit reflectance in the red colour band may increase chromatic contrast with leaves, and allow visually-oriented birds to identify and locate ripe fruits. These results confirm previous studies reporting that mammals exert weaker selective pressure for visual signals than birds^17^, and support previous findings that other sensory trajectories, such as touch or olfaction, are critical to some mammal-plant interactions^13–16,40–42^. While limited to Africa, our results are in agreement with the patterns reported in the Neotropics, where primate-dispersed species tend to be more dully coloured^18^ but more olfactorily conspicuous^14,25^. Moreover, the species included in this study originate from sites that differ in their climate, elevation and latitude (see methods). The fact that no significant site X syndrome interactions were found (Tables 1–4) indicates that the patterns recorded are consistent and are likely to repeat in other systems in the tropics.

In our study sites there are substantial differences within frugivorous mammal guilds: In Madagascar (ANP; RNP) the mammalian dispersers are lemurs^43^, of which many species are nocturnal or cathemeral, and most individuals are dichromatic^44^. In contrast, mammal-dispersed fruits in Uganda (KNP) are primarily dispersed by trichromatic monkeys and apes^45^. Yet surprisingly, we did not find significant interactions between site and dispersal mode, thus indicating that the effect of dispersers on fruit colour is uniform across study sites. This may indicate that even plant species that interact with fully trichromatic monkeys and apes experience relaxed selection pressures on fruit visual conspicuousness, possibly due to the ability of primates to rely on other senses, such as scent^14,16^.

At the same time, it should be noted that the questions we presented can, and should, be addressed through other lenses in future studies. First, while leaf reflectance is a useful and convenient proxy for various constraints and environmental conditions, it does not capture all factors which can affect fruit colour. For instance, fruit colour can be constrained by factors that are not expected to affect leaf colour such as floral pigmentation^4^. Yet another factor which could affect the strength of the selective pressures animals exert on fruit colour is the presence of redundant non-visual cues^15,16,23,25,46^. While some data regarding olfactory properties of the fruits used here are available^15,16,40,47^, they are not sufficiently standardized to be used as a control variable here. Finally, while the Brownian motion evolutionary models we used are a robust approach to control for the non-independence structure of any comparative dataset, the Brownian motion model is somewhat simplistic in its assumptions. Use of different models of evolution in future studies may shed more light on the dynamics behind the evolution of the patterns reported here.

In conclusion, our results confirm that fruit colour is largely independent of phylogeny and that it is affected by both abiotic factors and interaction with seed-dispersing animals. They join several recent studies which demonstrated the malleability of fruits to frugivore sensory and feeding ecology^17,20,21^. Yet they also highlight the importance of the multivariate approach which recognises that fruit colour is shaped by multidirectional selection pressures, and that only by considering them in concert can the effect of each be isolated and understood.

## Methods

### Study sites and sample collection

Ripe fruits and mature leaves of 97 species were collected opportunistically from three different protected areas. Fruits and leaves of 26 species were collected from Ankarafantsika National Park (ANP), Madagascar, between January and December 2012. Fruits and leaves of 36 species were collected from Ranomafana National Park (RNP), Madagascar, between Oct 2016 and Sep 2017, and of 35 species in Kibale National Park (KNP), Uganda, between May 2015 and December 2016. All three study sites host mammal and bird frugivores and plant species that either specialise on seed dispersal by one of them or by a combination of both. In ANP and RNP the majority of frugivores are lemurs – an endemic group of primates in which all or at least most individuals in all species are dichromats and the rest are trichromats, i.e. they can distinguish between red and green^48–50^. KNP supports a diverse community of fully trichromatic primates and largely tetrachromatic birds^45,51^. The three sites differ in many other ways. RNP and KNP are both montane rainforests (elevation: 900–1500 and 1100–1590 m; annual rainfall: 2300–4000, 1537 mm, respectively)^52–55^ whereas ANP, located in north-western Madagascar, is drier (1660 mm)^56,57^ and on lower elevation (100–170 m). The sites are located at different latitudes, with KNP closest to the equator (0°13′ N)^58^, and ANP (16°19′ S)^57^ and RNP (21°16′ S)^52^ further. Only fruits for which both fruit and leaf measurements were available, and which could be reliably assigned to disperser mode and genus were included in the analysis. Species were collected opportunistically and roughly represent the distribution of dispersal syndromes in the systems.

To quantify fruit and leaf colour, we measured the reflectance spectra of ripe fruits and leaves (1–10 individuals per species) relative to a Spectralon white reflectance standard (Labsphere). In ANP and KNP, we used a Jaz portable spectrometer and a PX-2 pulsed xenon lamp (Ocean Optics Inc) emitting a D-65 light source, with optical probes fixed at a 45 degree angle. In RNP, we measured reflectance using the same reflectance standard, light source and sampling parameters, with a USB2000 + UV-VIS miniature fibre optics spectrometer (Ocean Optics). Both spectrometers have gratings optimised to sample reflectance between 300–700 nm. For the analysed range, we obtained 1140 ± 2.40 (mean ± sd) values for each sample, which means that the average resolution for the measurement of the reflectance was 0.351 nm. In all cases where multiple individuals of the same species were sampled, we calculated the mean reflectance at each nm and used the resultant average to represent that species. We then standardised the reflectance so that the sum of all values between 300 nm and 700 nm was 1 (i.e., the total reflectance for each species for the analysed ranges was set to 1.

We assigned disperser mode to each fruit species based on personal observations and data available in the literature (Table S1). We classified species to three dispersal categories: birds (species which rely solely on birds for seed dispersal), mammals (species which are only consumed by mammals), and mixed (species dispersed by both birds and mammals).

### Analysis

For each species in the dataset we calculated the sum of relative reflectance in ripe fruits and mature leaves in four 100 nm reflectance bands: 300–400, 400–500, 500–600, and 600–700 nm. These bands broadly correspond to reflectance in the human colour categories of UV, blue, green and red. We treated reflectance in fruits at each of the four bands as a separate response variable that can be affected by either phylogeny, dispersal syndrome, or leaf colour. To estimate the importance of phylogeny, we used a published phylogeny^59^ exported using Phylomatic and then calculated Pagel’s Lambda^60^ using the function ‘phylosig’ from the phytools package v0.6–44^61^ in R v3.4.3. For each of the four colour bands, we used a likelihood ratio test (by setting the argument ‘test = TRUE’) to examine whether lambda is significantly different from 0, which implies some degree of phylogenetic signal. To estimate the relationship between fruit colour in each of these bands and disperser mode, leaf colour and phylogeny, we calculated Phylogenetic Generalised Least Squares models (PGLS) with a Brownian correlation structure using the function ‘gls’ from the R package nlme v3.1–131^62^. To account for variance that may originate from differences between the study sites, we included study site as a control factor in the models. Furthermore, we included the interaction between dispersal syndrome and site to account for the possibilities that the effect of different dispersers on fruit colour differs depending on geographic location. The general model formula was:

$$\begin{array}{c}Fruit\,reflectanc{e}_{i}\sim leaf.reflectanc{e}_{i}+dispersal.syndrome:study.site\\ \,\,\,\,\,\,\,+\,dispersal.syndrome+study.site\end{array}$$

We ran four different models, one for each of the colour bands with respective ripe fruit colour reflectance as response variable (*Fruit reflectance*_*i*_ with i = UV, Blue, Green, or Red), to identify the effects of each of the predictor variables independent of phylogeny. All continuous variables (reflectance of fruits and leaves) were square-root transformed to comply with the model’s assumptions. To establish the significance of full models, we compared the full models (containing all variables) with null models (excluding all independent variables) computing likelihood ratio tests using the function ‘anova’. To establish p-values for each of the predictor variables, we used the drop1 function (with the argument “test = ‘chisq’”), which excludes individual terms from the model and compares the resulting nested models to the full model using a chi-square test. Because in none of the models, the interaction between syndrome and site was significant (i.e., p < 0.05; see Tables 1–4), we excluded the interactions from the models and used the drop1 again to establish p-values for the main effects of syndrome and site, which were before included into the interaction. Thus, the effect of site on fruit colour in these models reflects differences between the sites which are independent of dispersal syndrome, phylogeny and leaf reflectance. As a result, they primarily reflect other unknown differences between the sites and were considered a control factor which allowed a more accurate comparison of the sites. Only in case the entire variable for dispersal syndrome was significant, we conducted pairwise comparisons between the three dispersal syndrome categories using the Tukey method as implemented in the function ‘emmeans’ from the package emmeans^63^. We used qq-plots and histograms of residuals, and scatterplots showing residuals against model fitted values to verify the model assumptions.

## Electronic supplementary material

Supplementary Dataset 1

## Data Availability

All data used for the analyses presented in this manuscript are available as online supplementary information.
