# Supplementary material for: The evolution of fruit colour: phylogeny, abiotic factors and the role of mutualists
Source: Sci Rep. 2018 Sep 24;8:14302. doi: 10.1038/s41598-018-32604-x (PMC6155155; doi:10.1038/s41598-018-32604-x)
Supplement: Supplementary file 1 — Supplementary Dataset 1 [file 41598_2018_32604_MOESM1_ESM.docx]

**Online supporting information**

**The evolution of fruit colour: phylogeny, abiotic factors and the role of mutualists**

Kim Valenta^1^, Urs Kalbitzer^2^, Diary Razafimadimby^3^, Patrick Omeja^4^, Manfred Ayasse^5^, Colin A. Chapman^2^, Omer Nevo^5*^.

1. Duke University, Department of Evolutionary Anthropology, 130 Science Dr. Durham, NC, 27708, USA

2. McGill University, McGill School of the Environment and Department of Anthropology, 3534 University Ave., Montreal, Quebec, Canada. H3A-2A7

3. Faculty of Sciences, Zoology and Animal Biodiversity, University of Antananarivo

4. Makerere University Biological Field Station, P.O. Box 907, Fort Portal, Uganda

5. University of Ulm, Institute of Evolutionary Ecology and Conservation Genomics

* Corresponding author: Omer Nevo, University of Ulm, Institute of Evolutionary Ecology and Conservation Genomics, Albert-Einstein-Allee 11, 89081 Ulm

omer.nevo@evolutionary-ecology.de

| **Family** | **Species** | **Dispersal**  **mode** | **Leaves (% reflectane in UV)** | **Leaves (% reflectane in blue)** | **Leaves (% reflectane in green)** | **Leaves (% reflectance in red)** | **Fruit (% reflectance in UV)** | **Fruit (% reflectance in blue)** | **Fruit (% reflectance in green)** | **Fruit (% reflectance in red)** | **Dispersal mode reference** |
| --- | --- | --- | --- | --- | --- | --- | --- | --- | --- | --- | --- |
| Ankarafantsika National Park | | | | | | | | | | | |
| Menispermaceae | *Anisocycle grandidieri* | B | 0.230 | 0.163 | 0.354 | 0.253 | 0.104 | 0.142 | 0.403 | 0.352 | ^1^ |
| Euphorbiaceae | *Antidesma petiolare* | BM | 0.106 | 0.150 | 0.474 | 0.270 | 0.040 | 0.017 | 0.021 | 0.922 | ^2,3^ |
| Meliaceae | *Asterotrichilia asterotricha* | M | 0.110 | 0.160 | 0.462 | 0.268 | 0.032 | 0.025 | 0.695 | 0.248 | ^3^ |
| Meliaceae | *Asterotrichilia spp* | M | 0.229 | 0.193 | 0.363 | 0.215 | 0.033 | 0.104 | 0.498 | 0.365 | ^3^ |
| Rhamnaceae | *Berchemia discolor* | M | 0.002 | 0.000 | 0.957 | 0.041 | 0.024 | 0.001 | 0.596 | 0.379 | ^4,5^ |
| Euphorbiaceae | *Bridellia pervilleana* | BM | 0.150 | 0.166 | 0.404 | 0.279 | 0.376 | 0.177 | 0.116 | 0.330 | ^1^ |
| Euphorbiaceae | *Croton spp* | B | 0.225 | 0.146 | 0.406 | 0.223 | 0.031 | 0.006 | 0.065 | 0.897 | ^1^ |
| Sapindaceae | *Doratoxylon chouxxi* | B | 0.197 | 0.154 | 0.367 | 0.281 | 0.127 | 0.042 | 0.024 | 0.806 | ^1^ |
| Elaeocarpaceae | *Elaeocarpus subserratus* | B | 0.167 | 0.153 | 0.405 | 0.275 | 0.085 | 0.116 | 0.318 | 0.481 | ^6^ |
| Rubiaceae | *Empogona ovalifolia* | BM | 0.135 | 0.095 | 0.523 | 0.247 | 0.293 | 0.067 | 0.033 | 0.607 | ^1^ |
| Clusiaceae | *Garcinia arenicola* | M | 0.073 | 0.078 | 0.523 | 0.326 | 0.077 | 0.148 | 0.363 | 0.412 | ^3^ |
| Rubiaceae | *Gardenia rutenbergiana* | M | 0.095 | 0.100 | 0.539 | 0.265 | 0.136 | 0.133 | 0.211 | 0.520 | ^3^ |
| Malvaceae | *Grewia madagascariensis* | B | 0.156 | 0.143 | 0.402 | 0.299 | 0.040 | 0.157 | 0.331 | 0.472 | ^6^ |
| Malvaceae | *Grewia spp* | BM | 0.267 | 0.183 | 0.337 | 0.213 | 0.133 | 0.173 | 0.283 | 0.411 | ^1^ |
| Rubiaceae | *Mapouria boinensis* | BM | 0.148 | 0.150 | 0.440 | 0.262 | 0.019 | 0.018 | 0.127 | 0.836 | ^1,5^ |
| Annonaceae | *Monanthotaxis valida* | BM | 0.245 | 0.167 | 0.384 | 0.204 | 0.042 | 0.046 | 0.319 | 0.593 | ^1,5,6^ |
| Oleaceae | *Noronhia spp* | BM | 0.059 | 0.110 | 0.560 | 0.270 | 0.011 | 0.015 | 0.088 | 0.885 | ^1^ |
| Apocynaceae | *Petchia spp* | B | 0.042 | 0.058 | 0.444 | 0.456 | 0.053 | 0.049 | 0.160 | 0.737 | ^6^ |
| Salvadoraceae | *Salvadora augustifolia* | B | 0.118 | 0.134 | 0.391 | 0.357 | 0.116 | 0.115 | 0.481 | 0.288 | ^6^ |
| Loganiaceae | *Strychnos decussata* | M | 0.079 | 0.107 | 0.516 | 0.298 | 0.131 | 0.129 | 0.296 | 0.444 | ^7^ |
| Loganiaceae | *Strychnos madagascariensis* | M | 0.068 | 0.092 | 0.602 | 0.238 | 0.005 | 0.025 | 0.333 | 0.637 | ^8,9^ |
| Loganiaceae | *Strychnos myrtoides* | BM | 0.066 | 0.129 | 0.534 | 0.270 | 0.048 | 0.025 | 0.162 | 0.766 | ^1,3^ |
| Loganiaceae | *Strychnos spinosa* | M | 0.047 | 0.101 | 0.446 | 0.406 | 0.028 | 0.032 | 0.342 | 0.598 | ^1^ |
| Combretaceae | *Terminalia trophophylla* | BM | 0.069 | 0.081 | 0.555 | 0.295 | 0.039 | 0.052 | 0.594 | 0.315 | ^1,3^ |
| Lamiaceae | *Vitex spp* | M | 0.108 | 0.117 | 0.491 | 0.284 | 0.234 | 0.172 | 0.264 | 0.330 | ^1^ |
| Rhamnaceae | *Ziziphus spp* | M | 0.122 | 0.123 | 0.470 | 0.285 | 0.081 | 0.057 | 0.255 | 0.607 | ^1^ |
| Kibale National Park | | | | | | | | | | | |
| Zingiberaceae | *Aframomum spp* | M | 0.292 | 0.134 | 0.397 | 0.177 | 0.077 | 0.031 | 0.104 | 0.788 | ^10^ |
| Sapotaceae | *Aningeria altissima* | BM | 0.194 | 0.119 | 0.438 | 0.250 | 0.220 | 0.166 | 0.363 | 0.251 | ^1,10^ |
| Salicaceae | *Casearia spp* | BM | 0.168 | 0.104 | 0.469 | 0.259 | 0.039 | 0.044 | 0.358 | 0.559 | ^1^ |
| Cannabaceae | *Celtis durandii* | BM | 0.218 | 0.119 | 0.465 | 0.197 | 0.020 | 0.003 | 0.281 | 0.696 | ^10,11^ |
| Apiaceae | *Centella spp* | B | 0.334 | 0.147 | 0.319 | 0.200 | 0.092 | 0.010 | 0.206 | 0.692 | ^1^ |
| Ulmaceae | *Chaetachme aristata* | M | 0.111 | 0.145 | 0.543 | 0.200 | 0.113 | 0.157 | 0.335 | 0.395 | ^10^ |
| Rutaceae | *Clausena anisata* | BM | 0.120 | 0.119 | 0.504 | 0.256 | 0.065 | 0.256 | 0.166 | 0.513 | ^1^ |
| Boraginaceae | *Cordia abyssinica* | BM | 0.217 | 0.165 | 0.332 | 0.286 | 0.077 | 0.053 | 0.350 | 0.519 | ^10,11^ |
| Boraginaceae | *Cordia millenii* | BM | 0.328 | 0.180 | 0.233 | 0.259 | 0.054 | 0.057 | 0.343 | 0.546 | ^10,11^ |
| Rubiaceae | *Craterispermum spp* | BM | 0.268 | 0.145 | 0.387 | 0.200 | 0.611 | 0.119 | 0.094 | 0.175 | ^1^ |
| Cucurbitaceae | *Cucumis spp* | M | 0.282 | 0.173 | 0.381 | 0.164 | 0.372 | 0.213 | 0.230 | 0.185 | ^12^ |
| Achariaceae | *Dasylepis eggelingi* | M | 0.356 | 0.129 | 0.337 | 0.178 | 0.156 | 0.120 | 0.190 | 0.534 | ^13^ |
| Capparaceae | *Euadenia eminens* | M | 0.241 | 0.105 | 0.455 | 0.199 | 0.224 | 0.138 | 0.245 | 0.393 | ^13^ |
| Rutaceae | *Fagaropsis angolensis* | BM | 0.272 | 0.141 | 0.379 | 0.207 | 0.310 | 0.089 | 0.106 | 0.495 | ^1,14^ |
| Moraceae | *Ficus brachylepsis* | BM | 0.314 | 0.194 | 0.295 | 0.197 | 0.169 | 0.122 | 0.300 | 0.409 | ^10,11^ |
| Moraceae | *Ficus cyathistipula* | BM | 0.251 | 0.149 | 0.415 | 0.184 | 0.207 | 0.169 | 0.312 | 0.312 | ^10,11^ |
| Moraceae | *Ficus dawei* | BM | 0.337 | 0.203 | 0.258 | 0.202 | 0.124 | 0.127 | 0.242 | 0.507 | ^10,11^ |
| Moraceae | *Ficus exasperata* | BM | 0.218 | 0.154 | 0.417 | 0.211 | 0.137 | 0.124 | 0.375 | 0.363 | ^10,11^ |
| Moraceae | *Ficus natalensis* | BM | 0.153 | 0.121 | 0.430 | 0.296 | 0.175 | 0.131 | 0.334 | 0.360 | ^10,11^ |
| Moraceae | *Ficus urcellaris* | BM | 0.136 | 0.139 | 0.508 | 0.217 | 0.022 | 0.019 | 0.258 | 0.702 | ^10,11^ |
| Moraceae | *Ficus vallis* | BM | 0.273 | 0.167 | 0.310 | 0.249 | 0.139 | 0.089 | 0.147 | 0.625 | ^10,11^ |
| Sapotaceae | *Mimusops bagshawei* | BM | 0.096 | 0.039 | 0.260 | 0.605 | 0.073 | 0.040 | 0.267 | 0.620 | ^10,11^ |
| Piperaceae | *Piper guineense* | BM | 0.243 | 0.177 | 0.385 | 0.195 | 0.045 | 0.013 | 0.160 | 0.782 | ^10,15^ |
| Apocynaceae | *Pleiocarpa spp* | M | 0.123 | 0.176 | 0.491 | 0.210 | 0.034 | 0.076 | 0.437 | 0.453 | ^14^ |
| Commelinaceae | *Polia spp* | B | 0.302 | 0.208 | 0.286 | 0.204 | 0.507 | 0.168 | 0.121 | 0.204 | ^16^ |
| Rosaceae | *Prunus africana* | BM | 0.205 | 0.143 | 0.441 | 0.212 | 0.192 | 0.112 | 0.104 | 0.592 | ^10,17^ |
| Anacardiaceae | *Pseudospondias microcarpa* | BM | 0.228 | 0.158 | 0.397 | 0.218 | 0.183 | 0.177 | 0.224 | 0.416 | ^10,18^ |
| Rubiaceae | *Rothmannia urcelliformis* | M | 0.290 | 0.159 | 0.337 | 0.214 | 0.217 | 0.127 | 0.381 | 0.275 | ^19,20^ |
| Solanaceae | *Solanum mauritianum* | BM | 0.202 | 0.202 | 0.347 | 0.249 | 0.119 | 0.056 | 0.170 | 0.655 | ^20,21^ |
| Olacaceae | *Strombosia spp* | BM | 0.329 | 0.109 | 0.389 | 0.173 | 0.109 | 0.136 | 0.463 | 0.293 | ^12,18^ |
| Myrtaceae | *Syzygium spp* | M | 0.127 | 0.137 | 0.492 | 0.244 | 0.166 | 0.218 | 0.277 | 0.340 | ^1^ |
| Apocynaceae | *Tabernaemontana conophrangia* | M | 0.316 | 0.177 | 0.328 | 0.179 | 0.227 | 0.146 | 0.306 | 0.321 | ^22^ |
| Rutaceae | *Teclea nobilis* | BM | 0.336 | 0.160 | 0.281 | 0.223 | 0.038 | 0.004 | 0.180 | 0.778 | ^10,11^ |
| Menispermaceae | *Tinospora spp* | B | 0.319 | 0.128 | 0.366 | 0.187 | 0.072 | 0.015 | 0.197 | 0.716 | ^1^ |
| Annonaceae | *Uvariopsis congensis* | BM | 0.309 | 0.161 | 0.336 | 0.194 | 0.125 | 0.091 | 0.152 | 0.632 | ^10,18^ |
| Ranomafana National Park | | | | | | | | | | | |
| Connaraceae | *Agelae pentagyna* | M | 0.173 | 0.006 | 0.503 | 0.318 | 0.090 | 0.158 | 0.408 | 0.345 | ^23,24^ |
| Sapindaceae | *Allophylus arboreus* | BM | 0.018 | 0.059 | 0.648 | 0.274 | 0.013 | 0.029 | 0.295 | 0.664 | ^24^ |
| Rubiaceae | *Chassalia ternifolia* | BM | 0.060 | 0.208 | 0.416 | 0.317 | 0.158 | 0.284 | 0.276 | 0.283 | ^23,24^ |
| Melastomataceae | *Clidemia hirta* | B | 0.141 | 0.127 | 0.409 | 0.324 | 0.170 | 0.213 | 0.304 | 0.313 | ^24,25^ |
| Rubiaceae | *Coptosperma spp* | M | 0.141 | 0.119 | 0.426 | 0.314 | 0.082 | 0.145 | 0.356 | 0.417 | ^24^ |
| Lauraceae | *Cryptocarya crassifolia* | BM | 0.002 | 0.013 | 0.771 | 0.215 | 0.022 | 0.028 | 0.085 | 0.864 | ^24^ |
| Lauraceae | *Cryptocarya spp* | M | 0.028 | 0.023 | 0.749 | 0.200 | 0.038 | 0.108 | 0.331 | 0.523 | ^23,24^ |
| Asparagaceae | *Dracaena spp* | B | 0.100 | 0.179 | 0.499 | 0.222 | 0.089 | 0.074 | 0.283 | 0.554 | ^24^ |
| Myrtaceae | *Eugenia spp* | M | 0.047 | 0.132 | 0.597 | 0.224 | 0.035 | 0.112 | 0.410 | 0.443 | ^23,24^ |
| Moraceae | *Ficus botryoides* | M | 0.096 | 0.155 | 0.491 | 0.258 | 0.103 | 0.182 | 0.321 | 0.393 | ^23,24^ |
| Moraceae | *Ficus lutea* | BM | 0.104 | 0.155 | 0.443 | 0.298 | 0.100 | 0.190 | 0.320 | 0.391 | ^23,24^ |
| Moraceae | *Ficus politoria* | BM | 0.035 | 0.065 | 0.608 | 0.293 | 0.069 | 0.092 | 0.212 | 0.627 | ^24,26,27^ |
| Moraceae | *Ficus reflexa* | BM | 0.276 | 0.078 | 0.303 | 0.343 | 0.352 | 0.098 | 0.207 | 0.343 | ^24^ |
| Moraceae | *Ficus tilliifolia* | M | 0.083 | 0.084 | 0.491 | 0.342 | 0.106 | 0.167 | 0.364 | 0.362 | ^23,24,28^ |
| Clusiaceae | *Garcinia goudotiana* | M | 0.127 | 0.000 | 0.718 | 0.155 | 0.021 | 0.142 | 0.432 | 0.406 | ^23,24^ |
| Clusiaceae | *Garcinia spp* | M | 0.165 | 0.141 | 0.336 | 0.359 | 0.141 | 0.196 | 0.322 | 0.340 | ^23,24^ |
| Euphorbiaceae | *Macaranga myriolepida* | B | 0.144 | 0.117 | 0.465 | 0.274 | 0.079 | 0.111 | 0.341 | 0.470 | ^23,24^ |
| Anacardiaceae | *Micronychia macrophylla* | M | 0.125 | 0.087 | 0.459 | 0.329 | 0.124 | 0.208 | 0.347 | 0.320 | ^23,24^ |
| Rubiaceae | *Mussaenda arcuata* | M | 0.058 | 0.142 | 0.540 | 0.260 | 0.059 | 0.059 | 0.432 | 0.450 | ^23,24^ |
| Oleaceae | *Noronhia incurvifolius* | M | 0.004 | 0.025 | 0.758 | 0.213 | 0.058 | 0.146 | 0.214 | 0.582 | ^24^ |
| Primulaceae | *Oncostemum botryoides* | M | 0.129 | 0.154 | 0.439 | 0.278 | 0.075 | 0.181 | 0.346 | 0.397 | ^23,24^ |
| Primulaceae | *Oncostemum nervosum* | B | 0.126 | 0.120 | 0.425 | 0.329 | 0.089 | 0.145 | 0.162 | 0.604 | ^23,24^ |
| Piperaceae | *Piper spp 1* | B | 0.063 | 0.154 | 0.528 | 0.254 | 0.087 | 0.063 | 0.184 | 0.666 | ^23,24^ |
| Piperaceae | *Piper spp 2* | BM | 0.239 | 0.002 | 0.339 | 0.420 | 0.091 | 0.186 | 0.313 | 0.410 | ^24^ |
| Pittosporaceae | *Pittosporum pachyphyllum* | M | 0.019 | 0.018 | 0.724 | 0.240 | 0.035 | 0.036 | 0.382 | 0.546 | ^29^ |
| Araliaceae | *Polyscias tripinnata* | BM | 0.075 | 0.066 | 0.513 | 0.346 | 0.011 | 0.118 | 0.103 | 0.768 | ^29^ |
| Myrtaceae | *Psidium cattleianum* | M | 0.005 | 0.000 | 0.946 | 0.049 | 0.046 | 0.111 | 0.238 | 0.605 | ^23,24^ |
| Hypericaceae | *Psorospermum androsaemifolium* | M | 0.003 | 0.000 | 0.854 | 0.143 | 0.023 | 0.043 | 0.422 | 0.512 | ^23,24^ |
| Rubiaceae | *Psychotria spp* | BM | 0.185 | 0.137 | 0.377 | 0.300 | 0.108 | 0.136 | 0.203 | 0.553 | ^23,24^ |
| Rubiaceae | *Pyrostria spp* | M | 0.107 | 0.163 | 0.475 | 0.255 | 0.113 | 0.131 | 0.292 | 0.464 | ^23,24^ |
| Araliaceae | *Schefflera spp* | B | 0.021 | 0.046 | 0.696 | 0.236 | 0.140 | 0.177 | 0.233 | 0.450 | ^29^ |
| Myrtaceae | *Syzygium emirnese* | M | 0.038 | 0.204 | 0.404 | 0.354 | 0.144 | 0.094 | 0.090 | 0.673 | ^23,24^ |
| Myrtaceae | *Syzygium parkeri* | M | 0.000 | 0.000 | 0.806 | 0.194 | 0.038 | 0.089 | 0.118 | 0.755 | ^23,24^ |
| Sapindaceae | *Tina striata* | M | 0.046 | 0.137 | 0.567 | 0.250 | 0.073 | 0.144 | 0.415 | 0.368 | ^23,24^ |
| Cunoniaceae | *Weinmannia rutenbergii* | M | 0.004 | 0.060 | 0.631 | 0.304 | 0.048 | 0.204 | 0.411 | 0.337 | ^23,24^ |
| Rutaceae | *Zanthoxylum madagascariensis* | BM | 0.047 | 0.106 | 0.613 | 0.234 | 0.023 | 0.176 | 0.441 | 0.360 | ^23^ |

**Supplementary Table 1:** Percent reflectance in leaves and ripe fruits in the UV (300-400nm), blue (400-500nm), green (500-600nm) and red (600-700nm) spectral bands. Dispersal mode: B = bird dispersed, M = mammal dispersed, BM = mixed dispersal.

1 Valenta, K. *Unpublished data*.

2 Simmen, B. *et al.* in *Ringtailed lemur biology* 55-68 (Springer, 2006).

3 Valenta, K. *et al.* Sensory integration during foraging: the importance of fruit hardness, colour, and odour to brown lemurs. *Behav. Ecol. Sociobiol.* **69**, 1855-1865 (2015).

4 Dudley, J. P. Seed dispersal by elephants in semiarid woodland habitats of Hwange National Park, Zimbabwe. *Biotropica* **32**, 556-561 (2000).

5 Sato, H. Frugivory and seed dispersal by brown lemurs in a Malagasy tropical dry forest. *Biotropica* **44**, 479-488 (2012).

6 Valenta, K. *et al.* Colour and odour drive fruit selection and seed dispersal by mouse lemurs. *Scientific Reports* **3**, 1-5 (2013).

7 Ganzhorn, J. U., Fietz, J., Rakotovao, E., Schwab, D. & Zinner, D. Lemurs and the regeneration of dry deciduous forest in Madagascar. *Conserv. Biol.* **13**, 794-804 (1999).

8 Sato, H. Seasonal fruiting and seed dispersal by the brown lemur in a tropical dry forest, north-western Madagascar. *J. Trop. Ecol.* **29**, 61-69 (2013).

9 Dausmann, K., GLOS, J., LINSENMAIR, K. & GANZHORN, J. Improved recruitment of a lemur-dispersed tree in Malagasy dry forests after the demise of vertebrates in forest fragments. *Oecologia* **157**, 307-316 (2008).

10 Potts, K. B., Watts, D. P. & Wrangham, R. W. Comparative feeding ecology of two communities of chimpanzees (Pan troglodytes) in Kibale National Park, Uganda. *Int. J. Primatol.* **32**, 669-690 (2011).

11 Obua, J. *The influence of fruit profiles on avian feeding strategies of the Kibale Forest Reserve, Uganda* MSc thesis, Makerere University, (1992).

12 Chapman, C. A. *Unpublished data*.

13 Bortolamiol, S. *et al.* Suitable habitats for endangered frugivorous mammals: small-scale comparison, regeneration forest and chimpanzee density in Kibale National Park, Uganda. *PLOS One* **9**, e102177 (2014).

14 Kagoro-Rugunda, G. & Hashimoto, C. Fruit phenology of tree species and chimpanzees’ choice of consumption in Kalinzu Forest Reserve, Uganda. *Open Journal of Ecology* **5**, 477 (2015).

15 Flörchinger, M., Braun, J., Böhning-Gaese, K. & Schaefer, H. M. Fruit size, crop mass, and plant height explain differential fruit choice of primates and birds. *Oecologia* **164**, 151-161 (2010).

16 Vignolini, S. *et al.* Pointillist structural color in Pollia fruit. *Proceedings of the National Academy of Sciences* **109**, 15712-15715 (2012).

17 Farwig, N., Böhning-Gaese, K. & Bleher, B. Enhanced seed dispersal of Prunus africana in fragmented and disturbed forests? *Oecologia* **147**, 238-252 (2006).

18 Paul, J. R. *Patterns of seed dispersal by animals: influence on sapling composition in a tropical forest* MSc thesis, University of Florida, (2001).

19 Krief, S. *et al.* Bioactive properties of plant species ingested by chimpanzees (*Pan troglodytes schweinfurthii*) in the Kibale National Park, Uganda. *Am. J. Primatol.* **68**, 51-71 (2006).

20 Krief, S. *Métabolites secondaires des plantes et comportement animal: surveillance sanitaire et observations de l'alimentation des chimpanzés (Pan troglodytes schweinfurthii) en Ouganda. Activités biologiques et étude chimique de plantes consommées*, Museum national d'histoire naturelle-MNHN PARIS, (2003).

21 Witkowski, E. & Garner, R. Seed production, seed bank dynamics, resprouting and long-term response to clearing of the alien invasive Solanum mauritianum in a temperate to subtropical riparian ecosystem. *S. Afr. J. Bot.* **74**, 476-484 (2008).

22 Potts, K. B., Chapman, C. A. & Lwanga, J. S. Floristic heterogeneity between forested sites in Kibale National Park, Uganda: insights into the fine‐scale determinants of density in a large‐bodied frugivorous primate. *J. Anim. Ecol.* **78**, 1269-1277 (2009).

23 Razafindratsima, O. H. & Dunham, A. E. Co‐fruiting plant species share similar fruit and seed traits while phylogenetic patterns vary through time. *J. Ecol.* **104**, 1789-1798 (2016).

24 Razafindratsima, O. H. F. E. *Nonrandom seed dispersal by lemur frugivores: mechanism, patterns and impacts*, Rice University, (2015).

25 Atsalis, S. Diet of the brown mouse lemur (Microcebus rufus) in Ranomafana National Park, Madagascar. *Int. J. Primatol.* **20**, 193-229 (1999).

26 Daru, B. H., Yessoufou, K., Nuttman, C. & Abalaka, J. A preliminary study of bird use of fig Ficus species in Amurum Forest Reserve, Nigeria. *Malimbus* **37**, 1-15 (2015).

27 Dowsett-Lemarie, F. Fruit choice and seed dissemination by birds and mammals in the evergreen forests of upland Malawi. (1988).

28 Martin, E. A., Ratsimisetra, L., Laloë, F. & Carrière, S. M. Conservation value for birds of traditionally managed isolated trees in an agricultural landscape of Madagascar. *Biodivers. Conserv.* **18**, 2719-2742 (2009).

29 Nevo, O. *Unpublished data*.
